# Mechanically activated zinc oxide enhances growth performance and protects against diarrhea with potentially regulating gut microbiota in weaned piglets

**DOI:** 10.1016/j.aninu.2026.03.002

**Published:** 2026-06-27

**Authors:** Zhijie Yuan, Jingqi Huang, Jinxin Guo, Bingbing Zhang, Weicheng Bei, Yong Wang, Yong Li, Hairui Xin, Weigang Xing, Yakuan Huang, Lvhui Sun, Zhangchao Deng

**Affiliations:** a National Key Laboratory of Agricultural Microbiology, Key Laboratory of Smart Farming Technology for Agricultural Animals of Ministry of Agriculture and Rural Affairs, Hubei Hongshan Laboratory, Frontiers Science Center for Animal Breeding and Sustainable Production, College of Animal Science and Technology, Huazhong Agricultural University, Wuhan 430070, Hubei, China; b Newhope Liuhe Co., Ltd., Beijing 100102, China; c The Cooperative Innovation Center for Sustainable Pig Production, College of Veterinary Medicine, Huazhong Agricultural University, Wuhan 430070, Hubei, China; d Yishi Biotechnology Co., Ltd., Shanghai 201700, China; e COFCO Feed Co., Ltd., Beijing 100102, China

**Keywords:** Zinc oxide, Intestinal health, Gut microbiota, Metabolomic, Tryptophan metabolism, Weaned piglet

## Abstract

High pharmacological doses of zinc oxide (ZnO) are widely used to control post-weaning diarrhea, but environmental pollution and potential adverse effects necessitate the search for effective low-dose alternatives. This study aimed to investigate the effects of dietary level of mechanically activated zinc oxide on growth performance and diarrhea in weaned piglets. A total of 1152 healthy weaned piglets (6.72 ± 0.63 kg) at 21 d of age were randomly assigned to six treatment groups with six replicates of 32 pigs per pen. Piglets received either a basal diet (BD), the BD supplemented with 100, 200, 400, or 600 mg Zn/kg mechanically activated zinc oxide (100 Zn, 200 Zn, 400 Zn, and 600 Zn), or the BD supplemented with 1600 mg Zn/kg conventional ZnO (1600 Zn) over a 28-d feeding period. Compared with the BD group, dietary 400 Zn supplementation significantly increased average daily gain during d 1 to 14, and decreased the feed/gain ratio during d 1 to 14 and d 1 to 28 (*P* < 0.05). Additionally, dietary 400 Zn supplementation consistently decreased the diarrhea rate regardless of the experimental period (*P* = 0.001). Notably, these effects are comparable to those observed with 1600 Zn treatment. Moreover, compared to 1600 Zn group, 400 or 600 Zn supplementation significantly reduced interleukin-6 (IL-6) content and diamine oxidase (DAO) activity, as well as significantly increased superoxide dismutase (SOD) activity (*P* < 0.05) in serum at d 28. Conversely, no significant differences were observed in serum malondialdehyde (MDA) and interleukin-1β (IL-1β) concentrations between the 400 or 600 Zn groups and the 1600 Zn group throughout the experimental period (*P* > 0.05). Further gut microbiome and serum metabolomic analysis found that the abundances of *Lactobacillus*, *Ligilactobacillus*, and *Roseburia* increased and tryptophan metabolism pathway was enriched by 400 Zn supplementation. Furthermore, the differential metabolites involved in tryptophan metabolism significantly correlated with most of differential genera. In conclusion, dietary supplementation with mechanically activated zinc oxide at 400 mg Zn/kg could exerted a certain positive effect on the growth performance of weaned piglets, which was comparable to or even superior to that of 1600 mg Zn/kg ZnO, indicating that mechanically activated zinc oxide could serve as an effective alternative to high-dose ZnO used in weaned piglets.

## 1 Introduction

Early weaning is a common management practice in modern swine production, offering several advantages such as improved reproductive efficiency of sows, enhanced growth performance and carcass quality of piglets, and reduced transmission of infectious diseases (Deng et al., 2024). Mounting evidence suggests that early weaning induces oxidative stress, characterized by excessive production of reactive oxygen species (ROS), which disrupts redox homeostasis and damages the intestinal epithelium (Deng et al., 2025a; Li et al., 2024; Tang et al., 2024). Oxidative injury compromises the intestinal barrier, increases mucosal permeability, and promotes epithelial cell apoptosis, all of which contribute to impaired intestinal function and higher susceptibility to post-weaning diarrhea (Han et al., 2024b; Shen et al., 2025; Zhang et al., 2020). Additionally, the abrupt dietary change is closely associated with gut microbiota dysbiosis, which is considered a key factor underlying the onset of weaning-associated diarrhea in piglets (Fang et al., 2025; Wu et al., 2026). With the progressive enforcement of antibiotic bans in animal husbandry, the identification and development of effective antibiotic alternatives to protect gut health and alleviate weaning stress in piglets have become an urgent priority (Ming et al., 2024).

Pharmacological doses of zinc oxide (ZnO) have long been used as an economical and effective strategy to control post-weaning diarrhea and promote growth in piglets. Numerous studies have confirmed its ability to improve intestinal morphology, reduce diarrhea incidence, and enhance feed conversion efficiency (Park et al., 2024; Schokker et al., 2023; Yi et al., 2023). Mechanistically, this antimicrobial efficacy is primarily attributed to the generation of ROS and the release of zinc ions (Zn^2+^), which disrupt bacterial cell membranes and oxidative homeostasis (Jiang et al., 2016; Sun et al., 2022). As a result, high-dose ZnO has been widely recognized as a viable substitute for in-feed antibiotics (Song et al., 2025; Xu et al., 2025; Yu et al., 2017). However, the therapeutic efficiency of conventional ZnO is limited by its low specific surface area; thus, high dosages are typically required to ensure sufficient contact with enteric pathogens. Consequently, prolonged supplementation with excessive ZnO poses serious risks, including environmental zinc accumulation, promotion of bacterial resistance, antagonism of other trace mineral absorption, and potential toxicity in piglets (Su et al., 2023; Zhe et al., 2021). In response to these concerns, novel forms of zinc with enhanced bioavailability and lower required dosages are being explored. For instance, nano-sized ZnO and ZnO quantum dots possess unique physicochemical characteristics that strengthen antimicrobial properties and bioavailability (Kociova et al., 2020; Shi et al., 2025; Wang et al., 2018). In addition, several organic derivatives, including Zn–polysaccharide conjugates and coated Zn formulations, are designed to enhance intestinal stability and facilitate more efficient delivery of zinc to the target sites of action (Lei and Kim, 2018; Xie et al., 2021).

Mechanically activated zinc oxide is a physically activated, micronized ZnO produced using innovative eccentric vibratory milling technology. Distinct from conventional micronization, this process not only reduces particle size to a diameter of less than 140 μm and substantially increases specific surface area but also stores additional energy within the particles (Peng et al., 2011). The primary advantage of this modification is the significant enhancement of physicochemical reactivity: the activation process induces a change in the electrical properties of the particles, which enhances their electrostatic attachment to bacterial cell membranes (Padmavathy and Vijayaraghavan, 2008). Furthermore, the improved solubility and high reactivity facilitate the generation of ROS upon contact with pathogens such as *Escherichia coli* (Barreto et al., 2017; Su et al., 2023; Wu et al., 2025). Based on these unique properties, it was hypothesized that the enhanced bioavailability and antimicrobial activity of mechanically activated zinc oxide would allow for a reduced dietary inclusion rate, effectively substituting for pharmacological doses of conventional ZnO to maintain growth performance and gut health. To test this hypothesis, the present study was conducted to evaluate the optimal inclusion level of mechanically activated zinc oxide in weaned piglets and to investigate its mechanisms in mitigating weaning-associated stress.

## 2 Materials and methods

### 2.1 Animal ethics statement

The animal experimental protocol was approved by the Animal Care and Use Committee of Huazhong Agricultural University (approval number HZAUSW-2025 to 0088).

### 2.2 Experimental design

A total of 1152 healthy Pig Improvement Company (PIC, Hendersonville, TN, USA) weaned piglets (6.72 ± 0.63 kg) at 21 d of age were randomly assigned to six treatment groups. Each group consisted of six replicates with 32 piglets per replicate. The control animals were fed a basal diet (BD) without additional zinc supplementation, while other animals in experimental groups were fed the BD with 100, 200, 400, or 600 mg Zn/kg mechanically activated zinc oxide (W. Schaumann GmbH & Co. KG, Eilsleben, Saxony-Anhalt, Germany), or 1600 mg Zn/kg of conventional ZnO (Ningxia Jingcheng Tianbao Technology Co., Ltd., Qingtongxia, Ningxia, China) supplementation. The supplementation dosages of mechanically activated zinc oxide were determined based on a preliminary experiment. The actual zinc contents of feed in the six groups were 98.5, 170, 235, 401, 625, and 1450 mg/kg, respectively. The mechanically activated zinc oxide additive consists of 95% ZnO with a diameter of less than 140 μm. The trial lasted for 28 d, during which pigs had ad libitum access to feed and water. The ingredients and nutrient levels of the BD is shown in Table 1.

**Table 1:** Ingredients and nutrient levels of the basal diet (as-fed basis, %).

Ingredients	Content	Nutrients^7^	Content
Corn	26.58	Digestible energy, MJ/kg	14.94
Whey powder	12.50	Dry matter	90.00
Broken rice	12.00	Crude protein	18.50
Extruded soybean	12.00	Ether extract	4.70
Extruded corn	12.00	Crude fiber	1.80
Soybean meal (46%)	9.18	Organic matter	94.90
Enzymatically hydrolyzed soybean protein (53%)	7.50	Calcium	0.66
Sucrose	4.00	Total phosphorus	0.60
Calcium phosphate dibasic	1.02		
L-Lysine hydrochloride (98.5%)	0.70		
Calcium formate	0.68		
Acidifier^1^	0.40		
DL-Methionine (98.5%)	0.35		
L-Threonine (98.5%)	0.34		
L-Tryptophan (98.5%)	0.09		
Mineral premix^2^	0.25		
Sodium chloride	0.12		
Enzyme preparation^3^	0.10		
Choline chloride (50%)	0.10		
Vitamin premix^4^	0.05		
Sweetener^5^ (contained ≥80% saccharin sodium)	0.02		
Phytase^6^ (20,000 U/g)	0.02		
Total	100.00		

1 Fengrunsuan III, Chengdu Fenglan Technology Co., Ltd., Chengdu, Sichuan, China.

2 The mineral premix provides per kg of the diet: Zn, 80 mg; Fe, 100 mg; Cu, 6 mg; Mn, 4 mg; I, 0.14 mg; Se, 0.3 mg.

3 FL511A, Wuhan Sunhy Biology Co., Ltd., Wuhan, Hubei, China.

4 The vitamin premix provides per kg of the diet: vitamin A, 2200 IU; vitamin D_3_, 220 IU; vitamin E, 16 IU; vitamin K_3_, 0.5 mg; thiamine, 1 mg; riboflavin, 3.5 mg; niacin, 30 mg; pantothenic acid, 10 mg; biotin, 0.05 mg; vitamin B_6_, 7 mg; vitamin B_12_, 17.5 μg.

5 Fengruntian II, Chengdu King-Tech Bio-tech Co., Ltd., Chengdu, Sichuan, China.

6 Qingdao Genyuan Biotechnology Group Co., Ltd., Qingdao, Shandong, China.

7 Nutrient levels were analyzed values, while digestible energy was calculated using data from the China Feed Database (2020), and organic matter was calculated as 100 minus ash content.

### 2.3 Growth performance, diarrhea incidence, and mortality

Feed intake was recorded daily and body weights were measured on d 1, 14, and 28 of the experiment. To assess diarrhea incidence, fecal consistency was visually assessed daily using a four-point scoring system: 0 = normal (firm, formed pellets); 1 = soft (well-formed but soft); 2 = mild diarrhea (thick, pasty, and unformed); and 3 = severe diarrhea (liquid or watery) (Fu et al., 2025). Piglets with a score of 2 or 3 were considered to have diarrhea. To ensure data accuracy in the group-housing system, identified piglets were marked to prevent duplicate counting on the same day. The diarrhea rate was calculated as follows:

Diarrhearate(%)=(Numberofpigletswithdiarrhea×Daysofdiarrhea)/(Totalnumberofpiglets×Experimentaldays)×100.

The number of dead piglets was recorded daily, and the mortality rate was calculated using the formula:

Mortalityrate(%)=(Totalnumberofdeadpiglets/Initialnumberofpiglets)×100.

In addition to diarrhea rate, average daily gain (ADG), average daily feed intake (ADFI), and feed/gain ratio were calculated accordingly.

### 2.4 Sample collection

On the d 14 and 28, six pigs closest to the average body weight within each of the 32 pens per treatment was selected for sampling. Blood samples were collected from the anterior vena cava into tubes without anticoagulant and allowed to clot at room temperature for 30 min. After centrifugation for 15 min, the separated serum was aliquoted and stored at −20 °C for subsequent biochemical analyses (Deng et al., 2024). The fecal samples were collected from the distal rectum to minimize potential interference from environmental microbiota for gut microbiota analysis. The collected samples were immediately placed into sterile cryovials, snap-frozen in liquid nitrogen, and subsequently stored at −80 °C.

### 2.5 Feed chemical composition analysis

Representative feed samples were collected and the nutritional levels in the basal diet according to the procedures of AOAC (2023). Dry matter (method 2001.12) was determined by drying samples in a forced-air oven (DHG-9140A, Shanghai Yiheng Scientific Instruments Co., Ltd., Shanghai, China) at 105 °C to a constant weight. Crude protein (method 954.01) was analyzed by the Kjeldahl method using an automatic Kjeldahl analyzer (Kjeltec 8400, FOSS Analytical A/S, Hillerød, Denmark). Ether extract (method 920.39) was determined by Soxhlet extraction using a fat analyzer (Soxtec 8000, Foss Analytical A/S, Hillerød, Denmark). Crude fiber (method 962.09) was analyzed through successive acid and alkali digestion using a fiber analyzer (Fibertec 8000, Foss Analytical A/S, Hillerød, Denmark). Crude ash (method 942.05) was measured by incineration at 550 °C in a muffle furnace (SX2-4-10, Shanghai Yiheng Scientific Instruments Co., Ltd., Shanghai, China). Calcium content (method 927.02) was determined via an atomic absorption spectrophotometer (AA-6880, Shimadzu Corporation, Kyoto, Japan), and phosphorus content (method 965.17) was measured photometrically using a UV–Vis spectrophotometer (UV-2600, Shimadzu Corporation, Kyoto, Japan). The value of digestible energy was calculated using data provided by the China Feed Database (2020). Organic matter content was calculated by subtracting the crude ash content from the dry matter content.

### 2.6 Determination of serum biochemistry, antioxidant, and inflammatory status

The activities of alanine aminotransferase (ALT), aspartate aminotransferase (AST), superoxide dismutase (SOD) and catalase (CAT), as well as the concentrations of blood urea nitrogen (BUN), creatinine (CRE), and malondialdehyde (MDA) were determined using corresponding commercial assay kits (C009-2-1, C010-2-1, A001-3, A007-1-1, C013-2-1, C011-2-1, and A003-1, respectively; Nanjing Jiancheng Bioengineering Research Institute, Nanjing, Jiangsu, China). The contents of lipopolysaccharides (LPS), diamine oxidase (DAO), interleukin-1 beta (IL-1β), interleukin-6 (IL-6), and interleukin-10 (IL-10) in serum were assayed by the commercially available assay kits (YT-37108O1, YT33277O1, YT-0422O1, YT-0425O1, and YT-0418O1; Jiangsu Yutong Biotechnology Co., Ltd., Yancheng, Jiangsu, China). All optical density (OD) values were measured with a monochromator-based multimode microplate reader (Spark, Tecan Trading AG, Männedorf, Switzerland).

### 2.7 16S rRNA gene sequencing and microbiome analysis

The gut microbiota of piglets was analyzed as previously described (Deng et al., 2025b; Yan et al., 2024). In brief, microbial DNA was isolated from fecal samples using the DNA Stool Mini Kit (Tiangen Biotech Co., Ltd., Beijing, China) following the manufacturer's instructions. The V3–V4 hypervariable regions of the 16S rRNA gene were amplified using the primers 338F (5′-ACTCCTACGGGAGGCAGCA-3′) and 806R (5′-GGACTACHVGGGTWVTAAT-3′). The sequencing library was established using the TruSeq Nano DNA LT Library Prep Kit (Illumina, Inc., San Diego, CA, USA), and all amplicons were sequenced using the Illumina Nova6000 platform in accordance with standard procedures. Raw sequences were quality filtered, denoised, and de-chimerized to avoid sequencing inaccuracy using the EasyAmplicon, which is a user-centric guide to perform amplicon sequencing data analysis (Yousuf et al., 2024). Taxonomy was assigned to amplicon sequence variants (ASVs) using the SILVA 138 database, and only ASVs detected in at least 50% of the group samples were included in the downstream analysis. The microbial taxonomic profiling, alpha diversity (including Shannon and Chao 1 indices), and principal coordinate analysis (PCoA) based on Bray–Curtis distance were conducted using the OmicStudio tools (Lyu et al., 2023). Linear discriminant analysis effect size (LEfSe) analysis, with an effect size threshold of 3, was employed to identify the differential abundance across groups. Spearman correlation analysis was performed, and correlations with coefficients <−0.4 or > 0.4 and adjusted *P*-values < 0.05 were considered significant (Cao et al., 2023). Group comparisons were determined using non-parametric test, and the false discovery rate (FDR) adjusted *P*-values were corrected by Benjamini-Hochberg method (Zhao et al., 2023).

### 2.8 Untargeted metabolomics

The serum untargeted metabolomic were conducted as previously described (Deng et al., 2023). Briefly, the serum samples were resuspended with prechilled 80% methanol, centrifuged at 15,000 × *g*, 4 °C for 10 min, and all supernatant concentrated to dry in vacuum. Samples were dissolved with 150 μL 80% methanol solution contain 4 μg/mL 2-chlorobenzalanine, and the supernatant was filtered through 0.22 μm membrane for liquid chromatography-mass spectrometry (LC-MS). The LC-MS analysis was performed using a Thermo Scientific Q Exactive HF-X mass spectrometer (Thermo Fisher Scientific Inc., Bremen, Germany) coupled with a vanquish ultra-high-performance liquid chromatography (UHPLC) system. Raw data were processed using Compound Discoverer 3.1 (Thermo Fisher Scientific Inc., Waltham, MA, USA) for peak alignment, picking, and annotation. Metabolites were identified by matching retention times and m/z values against the mzCloud and Kyoto Encyclopedia of Genes and Genomes (KEGG) databases. The partial least-squares discriminant analysis (PLS-DA) with variable importance in the projection (VIP) was used to find the differential metabolites between group. The differential metabolites were identified by *P*-value < 0.05 and VIP > 1. Metabolic pathways were enriched from the differential metabolites according to the KEGG pathway database (Zheng et al., 2024). The correlations among differential metabolites, serum parameters, and differential genera were analyzed by Spearman's rho correlation test via the OmicStudio tools, an online platform for data analysis (https://www.omicstudio.cn/tool).

### 2.9 Statistical analysis

In addition to the above-mentioned specific statistical methods of sequencing data, all data analyses were performed using SPSS version 19.0 (IBM Corp., Armonk, NY, USA). Data were analyzed by one-way ANOVA, followed by Duncan's multiple range test for multiple mean comparisons. The statistical model employed was a second-order polynomial regression, expressed as follows:

$$Y_{ij}=\beta_{0}+\beta_{1}X_{i}+\beta_{2}X_{i}^{2}+\varepsilon_{ij}\text{,}$$

where *Y*_*ij*_ is the observation *j* (*j* = 1-6) in treatment *i*, *β*_*0*_ is the overall intercept, *β*_*1*_ is the linear regression coefficient, *β*_*2*_ is the quadratic regression coefficient, *X*_*i*_ is the actual inclusion level of ZnO in diet *i*, and *ε*_*ij*_ is the random error with a mean of 0 and variance *σ*^*2*^. This model was used to test the linear and quadratic responses to increasing ZnO levels in the diets. Data are presented as mean and standard error of the mean (SEM), with a significance level of *P* < 0.05.

## 3 Results

### 3.1 Growth performance

As shown in Table 2, compared with the BD group, dietary supplementation with 400 or 600 Zn significantly increased ADG by 9.5%-27.8%, significantly reduced the F/G by 5.7%-12.4%, as well as significantly reduced diarrhea rate by 17.4%-25.1% during d 1 to 14 or d 1 to 28 (*P* < 0.05). Notably, these effects are comparable to those observed with 1600 Zn treatment. Additionally, no significant differences in mortality rate were observed among all groups throughout the trial (*P* > 0.05). Moreover, the ADG increased linearly (*P* < 0.05), while the F/G decreased linearly and quadratically (*P* < 0.05) as ZnO was supplemented during d 1 to 14 and d 1 to 28. The diarrhea rate decreased linearly (*P* = 0.007) as ZnO was supplemented during d 1 to 14.

**Table 2:** Effects of mechanically activated zinc oxide on the growth performance and diarrhea rate of piglets.

Items	Treatments^1^						SEM	*P*-value		
	BD	100 Zn	200 Zn	400 Zn	600 Zn	1600 Zn		T	L	Q
**d 1–14**										
ADG, g/d	176.07^c^	194.52^bc^	201.58^ab^	224.75^a^	208.38^ab^	222.20^a^	9.114	0.005	0.006	0.031
ADFI, g/d	253.98	255.88	267.33	279.28	265.04	274.16	8.230	0.245	0.158	0.264
Feed/gain ratio	1.45^a^	1.32^bc^	1.34^b^	1.24^c^	1.27^bc^	1.24^c^	0.032	0.001	0.001	0.004
Diarrhea rate, %	20.71^a^	19.99^a^	19.09^ab^	16.47^c^	17.10^bc^	12.42^d^	1.051	0.001	0.007	0.223
Mortality rate, %	0.52	0.00	0.52	0.00	0.00	0.52	0.351	0.663	0.298	0.243
**d 15–28**										
ADG, g/d	373.77	359.10	338.14	378.32	369.14	346.84	13.510	0.201	0.601	0.455
ADFI, g/d	519.61	507.59	488.04	522.34	502.71	469.07	14.130	0.105	0.985	0.569
Feed/gain ratio	1.40	1.42	1.45	1.38	1.37	1.35	0.032	0.140	0.399	0.642
Diarrhea rate, %	15.84^ab^	13.72^abc^	16.56^a^	10.86^cd^	13.45^abc^	7.95^d^	2.013	0.010	0.370	0.888
Mortality rate, %	0.52	0.57	0.00	0.00	0.00	0.52	0.402	0.732	0.159	0.132
**d 1–28**										
ADG, g/d	274.92	276.81	269.89	301.54	288.76	284.52	7.848	0.099	0.044	0.065
ADFI, g/d	386.79	381.74	377.68	400.81	383.87	371.62	8.973	0.318	0.511	0.355
Feed/gain	1.41^a^	1.38^a^	1.40^a^	1.33^b^	1.33^b^	1.31^b^	0.017	0.001	0.004	0.033
Diarrhea rate, %	18.27^a^	16.85^ab^	17.83^a^	13.66^b^	15.28^ab^	10.18^c^	1.389	0.001	0.092	0.541
Mortality rate, %	1.04	0.57	0.52	0.00	0.00	1.04	0.473	0.455	0.063	0.043

ADFI = average daily feed intake; ADG = average daily gain; T = treatment; L = linear; Q = quadratic; SEM = standard error of the mean.

Data are presented as means and SEM (*n* = 6). Means within the same row followed by different superscript letters are significantly different (*P* < 0.05).

1 Treatments: BD, basal diet; 100 Zn, BD + 100 mg Zn/kg mechanically activated zinc oxide ; 200 Zn, BD + 200 mg Zn/kg mechanically activated zinc oxide ; 400 Zn, BD + 400 mg Zn/kg mechanically activated zinc oxide; 600 Zn, BD + 600 mg Zn/kg mechanically activated zinc oxide; 1600 Zn, BD + 1600 mg Zn/kg conventional ZnO.

### 3.2 Serum biochemistry, antioxidant, and inflammatory status

As shown in Table 3, dietary zinc supplementation did not significantly affect serum ALT and AST activities, as well as BUN concentration, at both d 14 and 28 (*P* > 0.05). Similarly, no significant difference was observed in serum CRE concentration among the treatment groups at d 14 (*P* > 0.05). However, at d 28, serum CRE concentration was significantly increased in the 600 and 1600 Zn groups compared with the BD group (*P* = 0.023).

**Table 3:** Effects of mechanically activated zinc oxide on the serum biochemistry of piglets.

Items	Treatments^1^						SEM	*P*-value		
	BD	100 Zn	200 Zn	400 Zn	600 Zn	1600 Zn		T	L	Q
**d 14**										
ALT, U/L	31.86	28.69	35.41	29.73	29.28	32.01	2.582	0.442	0.523	0.498
AST, U/L	36.21	19.96	31.70	34.66	24.32	31.20	5.312	0.262	0.616	0.590
CRE, μmol/L	105.23	114.40	110.37	111.70	113.11	101.36	7.126	0.703	0.508	0.368
BUN, mmol/L	2.54	2.43	2.55	1.52	2.57	2.33	0.287	0.117	0.329	0.347
**d 28**										
ALT, U/L	46.20	58.69	48.00	51.80	51.71	53.98	8.483	0.867	0.894	0.957
AST, U/L	31.85	38.67	35.77	28.25	31.20	44.12	6.065	0.458	0.393	0.234
CRE, μmol/L	95.15^b^	86.92^b^	94.80^b^	100.63^ab^	113.27^a^	113.49^a^	7.678	0.023	0.074	0.214
BUN, mmol/L	2.31	2.28	2.51	2.06	2.44	3.07	0.346	0.446	0.769	0.434

ALT = alanine aminotransferase; AST = aspartate aminotransferase; BUN = blood urea nitrogen; CRE = creatinine; T = treatment; L = linear; Q = quadratic; SEM = standard error of the mean.

Data are presented as means and SEM (*n* = 6). Means within the same row followed by different superscript letters are significantly different (*P* < 0.05).

1 Treatments: BD, basal diet; 100 Zn, BD + 100 mg Zn/kg mechanically activated zinc oxide; 200 Zn, BD + 200 mg Zn/kg mechanically activated zinc oxide; 400 Zn, BD + 400 mg Zn/kg mechanically activated zinc oxide; 600 Zn, BD + 600 mg Zn/kg mechanically activated zinc oxide; 1600 Zn, BD + 1600 mg Zn/kg conventional ZnO.

As shown in Table 4, compared with the BD and 1600 Zn groups, dietary supplementation with 400 Zn significantly increased SOD activity at d 28 (*P* = 0.021), while MDA concentration showed a decreasing tendency (*P* = 0.080) up to the 400 Zn group at d 14. Meanwhile, dietary supplementation with 200 to 600 Zn significantly elevated CAT activity at both d 14 and 28 (*P* < 0.05), with the 400 Zn group showing the highest level. Moreover, the CAT activity increased linearly and quadratically (*P* < 0.05) as ZnO was supplemented at both d 14 and 28. The SOD activity increased linearly and quadratically (*P* < 0.05) as ZnO was supplemented at d 28, while MDA concentration decreased quadratically (*P* = 0.030) as ZnO was supplemented at d 14.

**Table 4:** Effects of mechanically activated zinc oxide on the serum antioxidant parameters of piglets.

Items	Treatments^1^						SEM	*P*-value		
	BD	100 Zn	200 Zn	400 Zn	600 Zn	1600 Zn		T	L	Q
**d 14**										
SOD, U/mL	21.64	24.71	23.31	24.13	22.90	21.15	1.155	0.249	0.464	0.263
CAT, U/mL	1.26^c^	1.67^bc^	2.22^ab^	2.90^a^	2.18^ab^	2.51^a^	0.293	0.004	0.007	0.021
MDA, nmol/mL	0.46	0.34	0.33	0.31	0.36	0.50	0.050	0.080	0.090	0.030
**d 28**										
SOD, U/mL	19.06^bc^	22.45^abc^	22.56^abc^	25.13^a^	22.93^ab^	18.76^c^	1.418	0.021	0.012	0.004
CAT, U/mL	1.99^b^	4.16^a^	3.71^a^	4.72^a^	4.64^a^	3.92^a^	0.591	0.012	0.004	0.007
MDA, nmol/mL	0.31	0.26	0.28	0.29	0.33	0.26	0.028	0.423	0.295	0.209

SOD = superoxide dismutase; CAT = catalase; MDA = malondialdehyde; T = treatment; L = linear; Q = quadratic; SEM = standard error of the mean.

Data are presented as means and SEM (*n* = 6). Means within the same row followed by different superscript letters are significantly different (*P* < 0.05).

1 Treatments: BD, basal diet; 100 Zn, BD + 100 mg Zn/kg mechanically activated zinc oxide; 200 Zn, BD + 200 mg Zn/kg mechanically activated zinc oxide; 400 Zn, BD + 400 mg Zn/kg mechanically activated zinc oxide; 600 Zn, BD + 600 mg Zn/kg mechanically activated zinc oxide; 1600 Zn, BD + 1600 mg Zn/kg conventional ZnO.

As shown in Table 5, dietary zinc supplementation did not significantly affect serum IL-1β concentration (*P* > 0.05). Compared with the BD group, serum concentrations of IL-6 in the 100 Zn group were significantly reduced (*P* = 0.001) at d 28, while IL-6 concentrations in the 600 and 1600 Zn groups significantly increased (*P* < 0.05) at both d 14 and 28. Compared to the 1600 Zn group, serum levels of IL-6 and DAO were significantly reduced (*P* < 0.05) by dietary supplementation with 400 or 600 Zn at d 28. Additionally, serum IL-10 concentration increased linearly (*P* = 0.049) with increasing doses of mechanically activated zinc oxide treatment on d 14; however, this trend was reversed on d 28. The IL-6 concentration increased linearly and quadratically (*P* < 0.05) as ZnO was supplemented at d 14, while DAO concentration decreased quadratically (*P* = 0.040) as ZnO was supplemented at d 28.

**Table 5:** Effects of mechanically activated zinc oxide on the serum inflammatory parameters of piglets.

Items	Treatments^1^						SEM	*P*-value		
	BD	100 Zn	200 Zn	400 Zn	600 Zn	1600 Zn		T	L	Q
**d 14**										
IL-1β, ng/L	28.77	33.78	31.02	33.55	35.52	34.30	2.112	0.269	0.065	0.119
IL-6, ng/L	699.02^b^	729.03^b^	789.94^ab^	776.73^ab^	850.86^a^	858.25^a^	31.680	0.008	0.004	0.028
IL-10, ng/L	132.33^d^	142.81^cd^	151.10^bc^	163.88^b^	163.02^b^	191.29^a^	11.196	0.002	0.049	0.300
LPS, ng/L	395.25	337.34	359.52	359.24	364.48	393.12	19.716	0.232	0.444	0.284
DAO, pg/mL	97.26	88.14	71.16	77.30	99.62	98.68	10.024	0.139	0.798	0.593
**d 28**										
IL-1β, ng/L	50.84	45.37	43.52	51.80	46.48	44.42	2.225	0.067	0.966	0.745
IL-6, ng/L	810.32^c^	706.41^d^	801.81^c^	815.70^c^	913.34^b^	1103.24^a^	29.716	0.001	0.051	0.880
IL-10, ng/L	248.05^a^	224.78^b^	214.20^b^	221.90^b^	207.77^b^	222.44^b^	6.625	0.006	0.001	0.002
LPS, ng/L	371.67	352.18	348.08	339.58	382.26	364.60	20.290	0.602	0.918	0.994
DAO, pg/mL	147.58^a^	110.63^bcd^	95.06^d^	97.42^cd^	120.06^bc^	129.65^ab^	10.608	0.001	0.065	0.040

IL-1β = interleukin-1 beta; IL-6 = interleukin-6; IL-10 = interleukin-10; LPS = lipopolysaccharides; DAO = diamine oxidase; T = treatment; L = linear; Q = quadratic; SEM = standard error of the mean.

Data are presented as means and SEM (*n* = 6). Means within the same row followed by different superscript letters are significantly different (*P* < 0.05).

1 Treatments: BD, basal diet; 100 Zn, BD + 100 mg Zn/kg mechanically activated zinc oxide; 200 Zn, BD + 200 mg Zn/kg mechanically activated zinc oxide; 400 Zn, BD + 400 mg Zn/kg mechanically activated zinc oxide; 600 Zn, BD + 600 mg Zn/kg mechanically activated zinc oxide; 1600 Zn, BD + 1600 mg Zn/kg conventional ZnO.

### 3.3 Gut microbiota

**Fig. 1:**
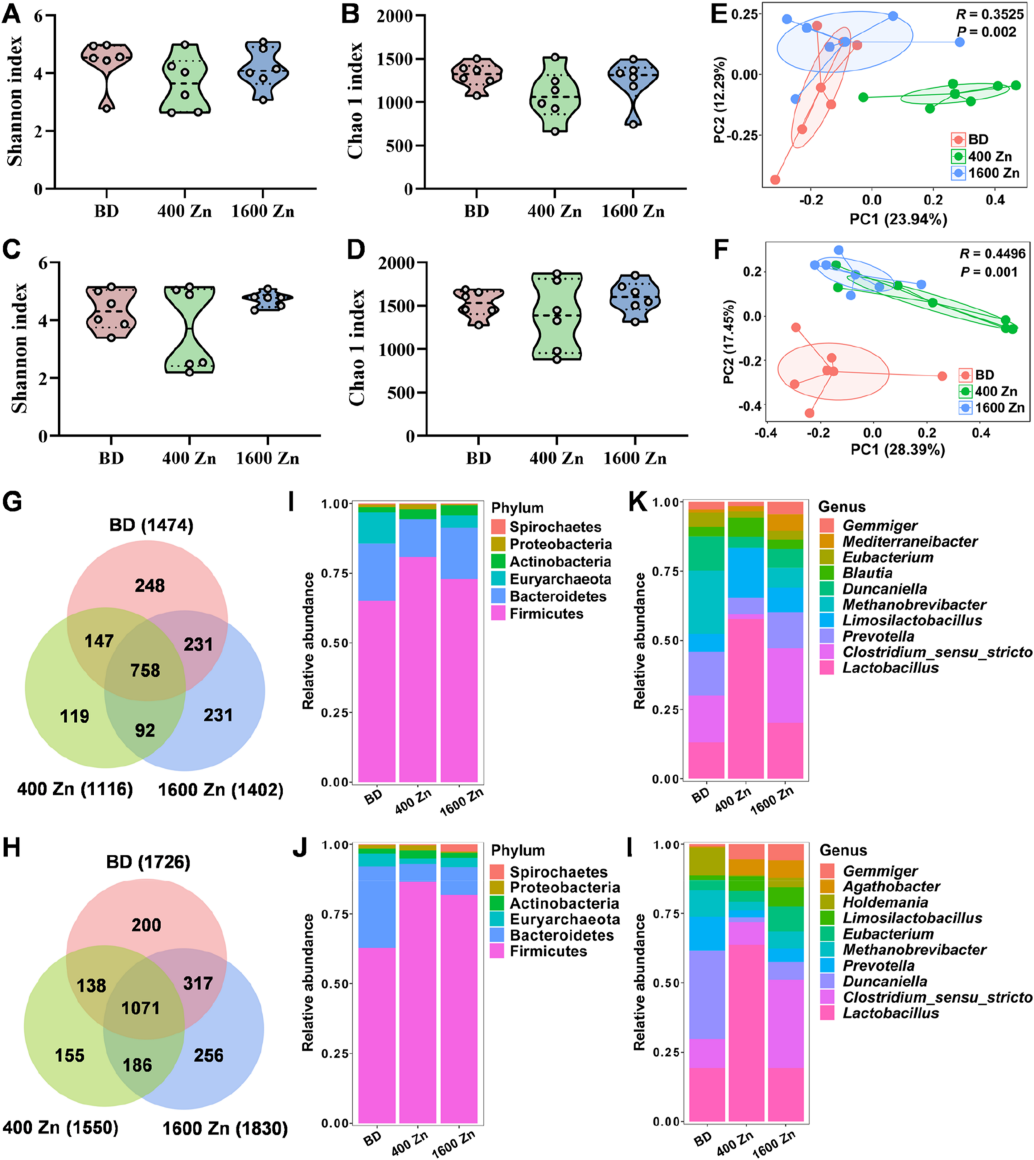
Microbial diversities and composition of the piglet gut microbiota. The microbial diversities were calculated by Shannon index (A, C), Chao 1 index (B, D) and principal coordinate analysis (PCoA) plots based on Bray-Curtis (E, F). Numbers of amplicon sequence variants (ASVs) (G, H), relative abundance of the top six phylum (I, J), and top ten genera (K, L) among the three groups. A, B, E, G, I, and K represent d 14, while C, D, F, H, J, and L represent d 28. BD, basal diet group; 400 Zn and 1600 Zn, BD supplemented with 400 mg Zn/kg mechanically activated zinc oxide and 1600 mg Zn/kg conventional ZnO, respectively. *n* = 6 per group. PC = principal component.

**Fig. 2:**
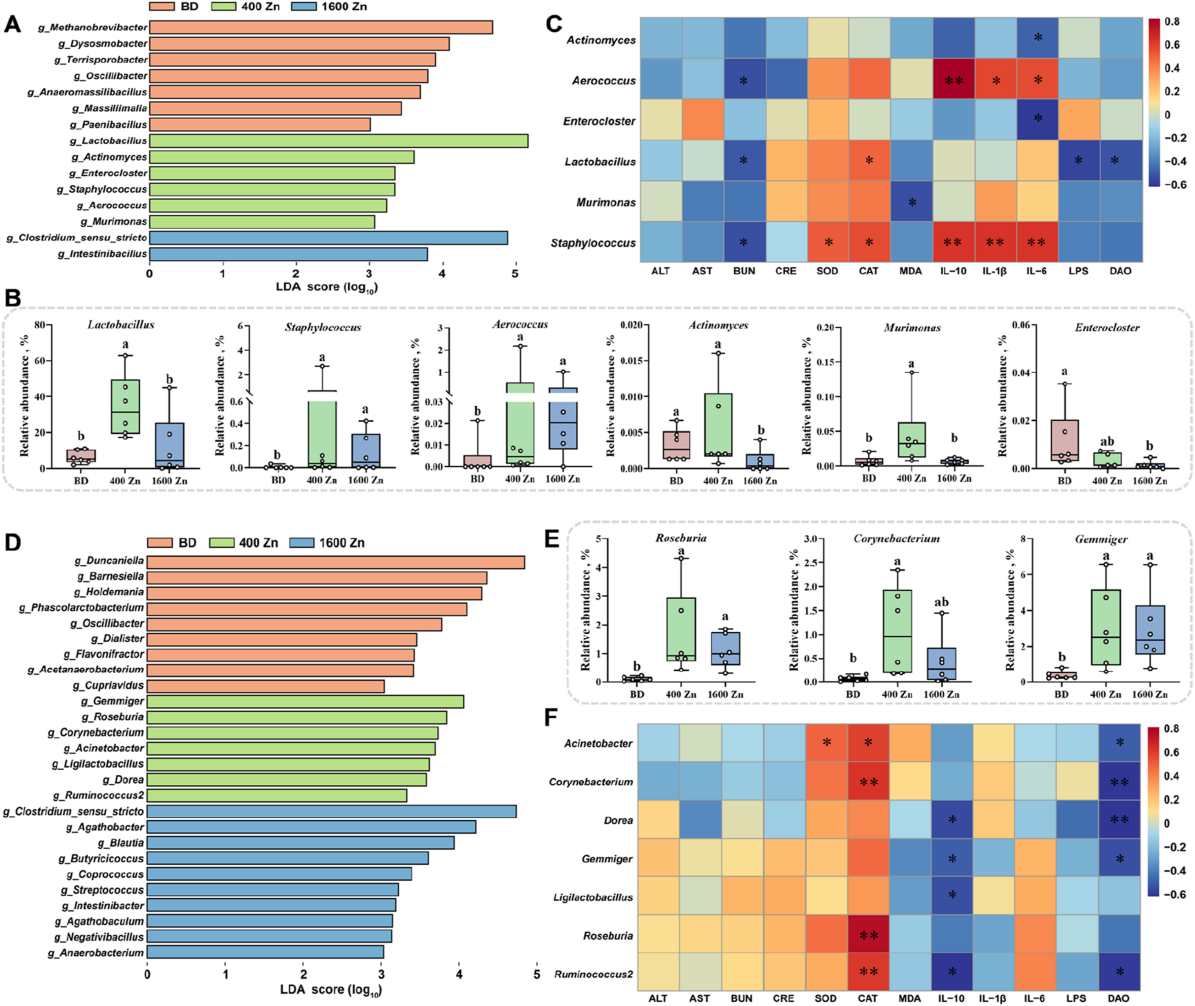
Identification and correlation analysis of differential bacteria in piglets. Differential enrichment of the bacterial genus was identified using linear discriminant analysis effect size (LEfSe) analysis (A, D). Relative abundance of seven differential genus enriched in 400 Zn group (B, E). Spearman correlation between serum biochemical parameters and the differentially expressed genus (C, F). Correlation coefficients and *P*-values were determined via Spearman's rho correlation test. A, B, and C represent d 14, while D, E, and F represent d 28. BD, basal diet group; 400 Zn and 1600 Zn, BD supplemented with 400 mg Zn/kg mechanically activated zinc oxide and 1600 mg Zn/kg conventional ZnO, respectively. ∗, *P* < 0.05; ∗∗, *P* < 0.01. Labeled means with different superscript letters are significantly different (*P* < 0.05). *n* = 6 per group. LDA = linear discriminant analysis; ALT = alanine aminotransferase; AST = aspartate aminotransferase; BUN = blood urea nitrogen; CRE = creatinine; SOD = superoxide dismutase; CAT = catalase; MDA = malondialdehyde; IL-10 = interleukin-10; IL-1β = interleukin-1 beta; IL-6 = interleukin-6; LPS = lipopolysaccharide; DAO = diamine oxidase.

A total of 36 fecal samples were subjected to 16S rRNA gene sequencing, and identified 2655 and 2868 ASVs at d 14 and 28, respectively (Tables S1 and S2). The results of gut microbial diversity showed no significant differences (*P* > 0.05) were observed in alpha diversity indices (Shannon index and Chao 1 index) among three groups at both d 14 and 28 (Fig. 1A–D). Further PCoA analysis revealed a significant separation (*P* < 0.05) among three groups on d 14 and 28 (Fig. 1E and F). Meanwhile, the three groups shared 758 ASVs on d 14 (Fig. 1G), while shared 1071 ASVs on d 28 (Fig. 2H). At the phylum level, Firmicutes is the highest abundant phylum, and its abundance increased after mechanically activated zinc oxide treatment (Fig. 1I and J). Moreover, dietary supplementation with 400 Zn exhibited greater changes at the genus level, particularly an increase in *Lactobacillus* compared to the BD group (Fig. 1K and L).

Further LEfSe analysis revealed 15 significantly different genera among groups at d 14 (Fig. 2A), and the relative abundances of *Lactobacillus*, *Staphylococcus*, *Aerococcus*, and *Murimonas* were significantly elevated (*P* < 0.05) in the 400 Zn group compared with the BD group (Fig. 2B). At d 28, a total of 31 significantly different genera were identified by LEfSe analysis (Fig. 2D), among which *Gemmiger*, *Roseburia*, *Corynebacterium*, *Ruminococcus2*, *Ligilactobacillus*, *Dorea*, and *Acinetobacter* were significantly enriched (*P* < 0.05) in the 400 Zn group (Fig. 2E and Fig. S1). Furthermore, Spearman correlation analysis showed that the abundance of these differential genera displayed significant correlations with several serum parameters (Fig. 2C and E). For example, at d 14, the abundance of *Staphylococcus* and *Aerococcus* were positively correlated (*R* > 0.55, *P* < 0.05) with IL-10, IL-1β, and IL-6 contents, while *Lactobacillus* abundance was negatively correlated (*R* < −0.49, *P* < 0.05) with BUN, LPS, and DAO contents (Fig. 2C). Additionally, at d 28, serum CAT activity had significant positive correlation (*R* > 0.57, *P* < 0.05) with the abundance of *Acinetobacter*, *Corynebacterium*, *Roseburia*, and *Ruminococcus2*, while IL-10 content and DAO activity had negative correlation (*R* < −0.47, *P* < 0.05) with abundance of *Dorea*, *Gemmiger*, and *Ruminococcus2* (Fig. 2E).

### 3.4 Serum metabolomics

**Fig. 3:**
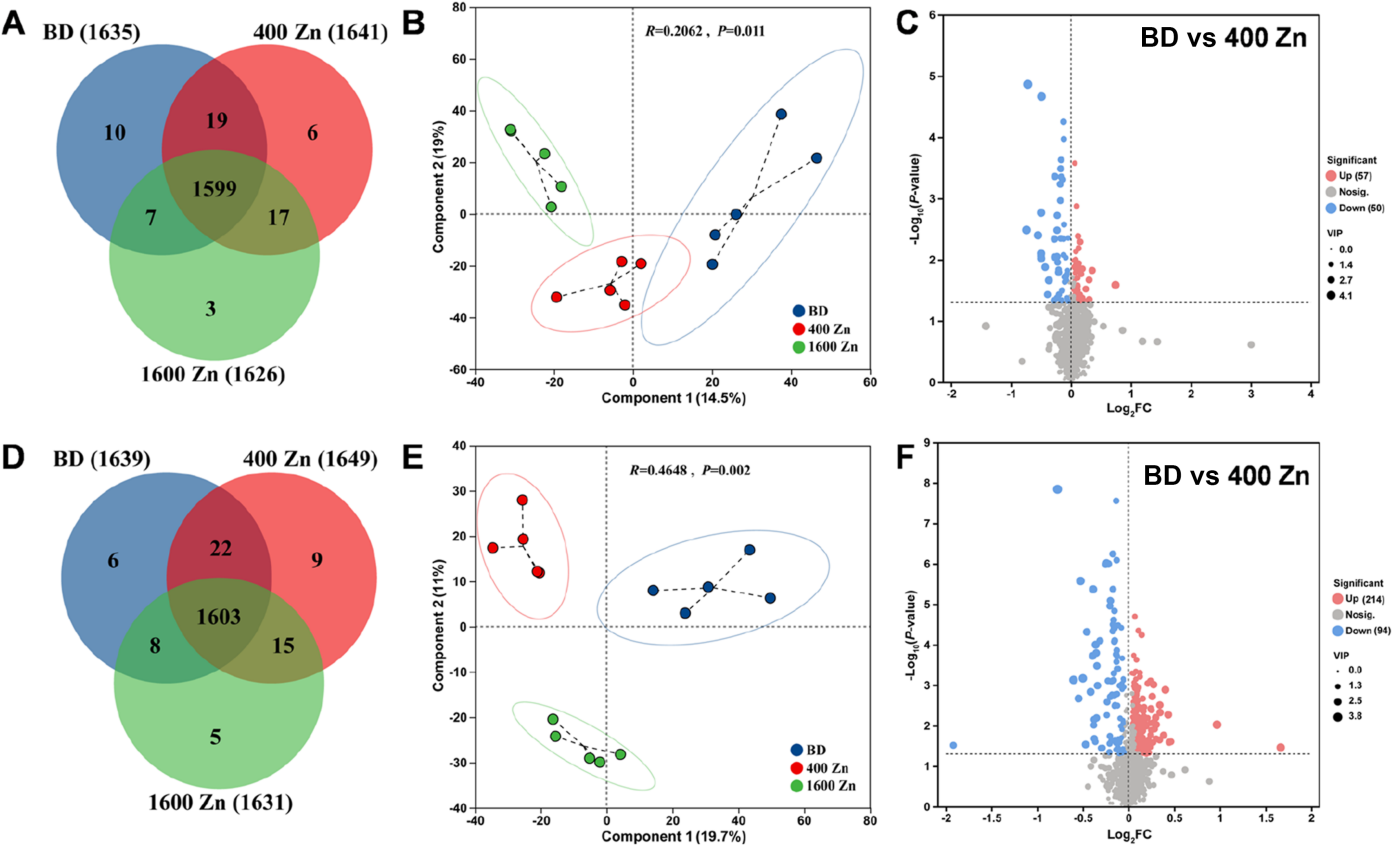
Serum metabolomics analysis of piglets. Numbers of identified serum metabolites in piglets among three groups. Partial least-squares discriminant analysis (PLS-DA) score plot of the serum metabolomic profiles. Differences were tested by analysis of similarities (ANOSIM). Volcano plot analysis of the differential metabolites between BD and 400 Zn groups. A, B, and C represent d 14, while D, E, and F represent d 28. BD, basal diet group; 400 Zn and 1600 Zn, BD supplemented with 400 mg Zn/kg mechanically activated zinc oxide and 1600 mg Zn/kg conventional ZnO, respectively. FC = fold change; VIP = variable importance in projection.

**Fig. 4:**
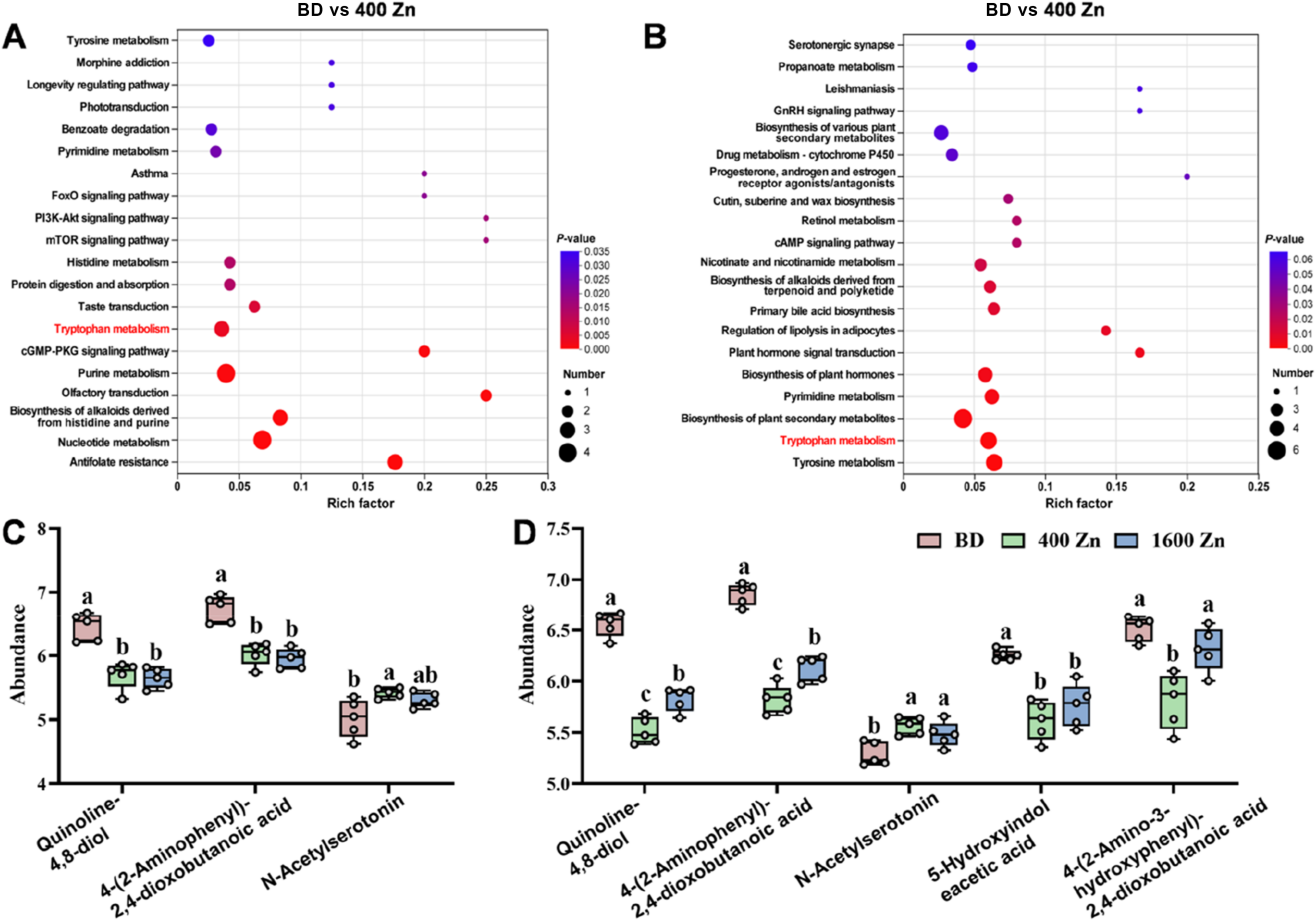
Identification of key differential metabolites in piglets. Kyoto Encyclopedia of Genes and Genomes (KEGG) pathway analysis of the serum differential metabolites between BD and 400 Zn groups in piglets at d 14 (A) and 28 (B). Abundance of differential metabolites involved in tryptophan metabolism in piglets at d 14 (C) and 28 (D). BD, basal diet group; 400 Zn and 1600 Zn, BD supplemented with 400 mg Zn/kg mechanically activated zinc oxide and 1600 mg Zn/kg conventional ZnO, respectively. Labeled means with different superscript letters are significantly different (*P* < 0.05). *n* = 5 per group. FoxO = forkhead box O; PI3K-Akt = phosphoinositide 3-kinase-protein kinase B; mTOR = mechanistic target of rapamycin; cGMP-PKG = cyclic guanosine monophosphate-protein kinase G; GnRH = gonadotropin-releasing hormone; cAMP = cyclic adenosine monophosphate.

A total of 1661 and 1668 metabolites were identified in the serum at d 14 and 28, respectively (Tables S3 and S4). Among these, 1599 and 1603 metabolites were shared among the three groups at d 14 and 28, respectively (Fig. 3A and D). Partial least squares discriminant analysis revealed that the metabolomic profiles were clearly separated among the three groups at both time points (Fig. 3B and E). Additionally, differentially expressed metabolites between each pair of groups were identified (Fig. 3C–F, and Fig. S2). Specifically, a total of 57 and 214 metabolites were significantly upregulated, while 50 and 94 were downregulated in the 400 Zn group compared with the BD group on d 14 and 28, respectively (Fig. 3C and F). Further KEGG pathway enrichment analysis revealed that tryptophan metabolism emerged as a key metabolic pathway following dietary treatment at both time points (Fig. 4A and B, and Fig. S3). Notably, several metabolites involved in tryptophan metabolism were altered following supplementation with 400 Zn. Specially, compared with the BD group, the concentrations of quinoline-4,8-diol and 4-(2-aminophenyl)-2,4-dioxobutanoic acid were significantly reduced (*P* < 0.05), while N-acetylserotonin significantly increased (*P* < 0.05) at both d 14 and 28 (Fig. 4C and D).

### 3.5 Integrated analysis of differential metabolites involved in tryptophan metabolism

**Fig. 5:**
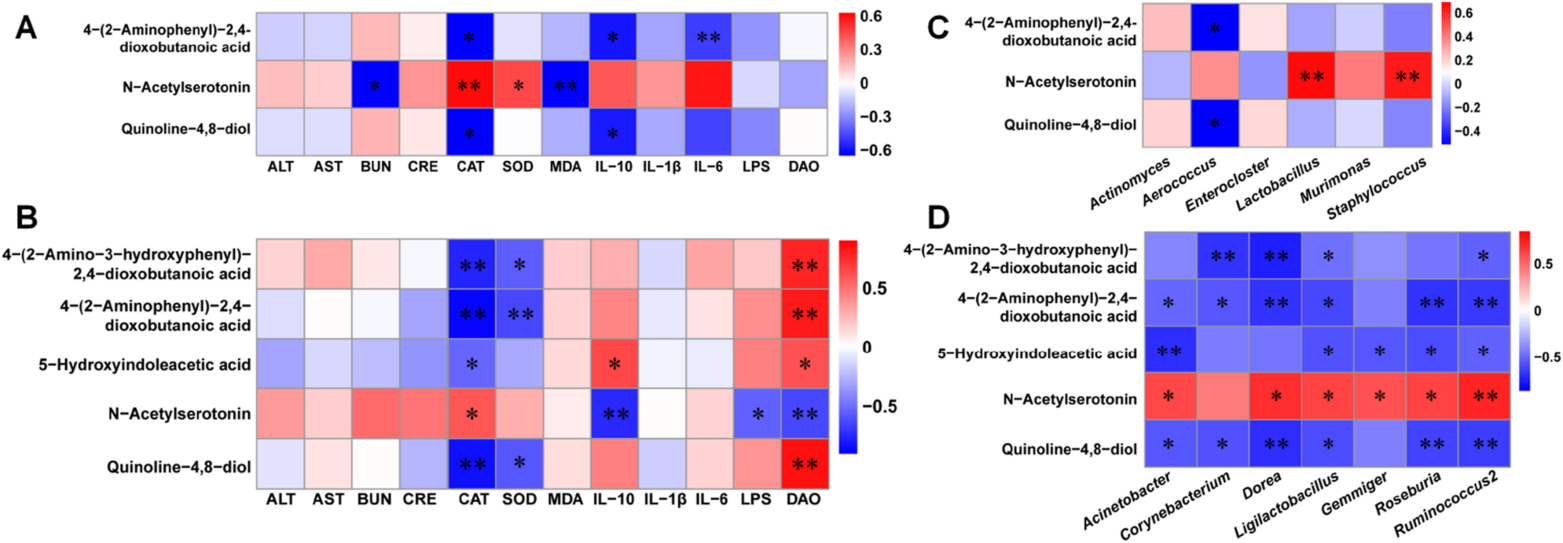
Integrated analysis of differential metabolites involved in tryptophan metabolism of piglets. Spearman correlation between differential metabolites and serum biochemical parameters of piglets at d 14 (A) and 28 (B). Spearman correlation between differential metabolites and bacterial genus at d 14 (C) and 28 (D). Correlation coefficients and *P*-values were determined via Spearman's rho correlation test. ∗, *P* < 0.05; ∗∗, *P* < 0.01. ALT = alanine aminotransferase; AST = aspartate aminotransferase; BUN = blood urea nitrogen; CRE = creatinine; SOD = superoxide dismutase; MDA = malondialdehyde; IL-10 = interleukin-10; IL-1β = interleukin-1 beta; IL-6 = interleukin-6; LPS = lipopolysaccharide; DAO = diamine oxidase.

Spearman correlation analysis of differential metabolites involved in tryptophan metabolism and serum parameters is shown in Fig. 5A and B. Specifically, serum CAT activity was significantly correlated (|r| > 0.52, *P* < 0.05) with all the differential metabolites involved in tryptophan metabolism at both d 14 and 28 (Fig. 5A and B). Additionally, serum DAO activity also showed positive correlation (*|r|* > 0.54, *P* < 0.05) with all these metabolites, expect for N-acetylserotonin, at d 28 (Fig. 5B). Further correlation analysis of these metabolites and differential microbes showed that quinoline-4,8-diol and 4-(2-aminophenyl)-2,4-dioxobutanoic acid were negatively correlated (*|r|* < −0.52, *P* < 0.05) with *Aerococcus*, while N-acetylserotonin showed significant positive correlations (|r| > 0.64, *P* < 0.01) with *Lactobacillus* and *Staphylococcus* on d 14 (Fig. 5C). Notably, only N-acetylserotonin was positively associated (*|r|* > 0.53, *P* < 0.05) with most differential genera, while other metabolites were negatively associated (*|r|* < −0.52, *P* < 0.05) with most differential genera at d 28 (Fig. 5D).

## 4 Discussion

This study demonstrated that dietary supplementation with mechanically activated zinc oxide significantly improved growth performance in weaned piglets. Specifically, supplementation with mechanically activated zinc oxide at levels above 400 mg Zn/kg increased ADG, as well as reduced F/G and diarrhea ratio at some experiment phase, effects comparable to those observed with 1600 mg Zn/kg ZnO. These findings support previous reports that ZnO supplementation benefits the growth performance of weaned piglets (Cui et al., 1997; Sun et al., 2022), and indicate that mechanically activated zinc oxide could serve as an effective alternative to high-dose ZnO in weaned piglet diets. Unlike conventional ZnO, which relies on high dietary concentrations to ensure sufficient pathogen contact, mechanically activated zinc oxide achieves comparable efficacy at a fraction of the dosage, highlighting its superior bioavailability.

Serum biochemical indicators, which reflect changes in tissue cell permeability and metabolism within the body, have been recognized as valuable biomarkers for assessing animal health status (Gao et al., 2024). Among them, AST and ALT are nonspecific enzymes in the blood that are present in many organs (Souza et al., 2025). Elevated levels of both indicators detected in the serum are indicative of liver damage (Lee et al., 2025). In the present study, dietary supplementation with 200 to 600 mg Zn/kg mechanically activated zinc oxide did not alter serum ALT and AST levels, indicating the safety of mechanically activated zinc oxide for the liver of piglet. Furthermore, serum BUN is an important measure of protein anabolism, with lower levels indicating greater protein synthesis (Jiang and Wu, 2025). In this study, 400 mg Zn/kg mechanically activated zinc oxide reduced the serum BUN content at d 14, suggesting that the addition of mechanically activated zinc oxide in the diet was beneficial to improve the utilization of protein and promote the growth of piglets. Thus, in short, dietary mechanically activated zinc oxide supplementation at 400 mg Zn/kg could help improve health status of weaned piglets, thereby improving growth performance.

Accumulating evidence suggests that redox homeostasis is a critical determinant of intestinal health after weaning, as oxidative stress acts as a key mediator of weaning-induced intestinal dysfunction (Degroote et al., 2020). In this study, supplementation with 400 mg Zn/kg mechanically activated zinc oxide significantly enhanced antioxidant enzyme activities, particularly CAT and SOD, while reducing the level of MDA. These findings suggest that, similar to other zinc-based interventions, mechanically activated zinc oxide may alleviate oxidative damage by enhancing endogenous antioxidant defenses (Jiang et al., 2023; Liu et al., 2024). This effect may be attributed to the high internal energy and increased specific surface area of mechanically activated zinc oxide, which improved its bioavailability and enhances reactivity with the intestinal mucosa, facilitating more effective interactions with epithelial cells and the mucosal immune system. In contrast, although 1600 mg Zn/kg ZnO is effective in reducing diarrhea, it is associated with concerns such as mineral accumulation, bacterial resistance, and environmental burden (Liu et al., 2025; Su et al., 2023; Wang et al., 2020, 2024b). Notably, compared to the 1600 mg Zn/kg ZnO group, the 400 mg Zn/kg mechanically activated zinc oxide treatment achieved comparable or even superior effects on antioxidant capacity. These results suggest that mechanically activated zinc oxide overcomes the low solubility limitation of conventional ZnO and could be an effective alternative to high-dose ZnO used in weaned piglets.

Gut microbiota plays a vital role in maintaining intestinal health; however, gut dysbiosis can lead to intestinal inflammation and diarrhea in weanling piglets (Ma et al., 2026). In the present study, microbial profiling showed that dietary supplementation with 400 mg Zn/kg mechanically activated zinc oxide exhibited a beneficial shift in gut microbiota composition, notably characterized by increased abundances of *Lactobacillus*, *Ligilactobacillus*, and *Roseburia*. *Lactobacillus* and *Ligilactobacillus* are classical probiotic genera capable of enhancing epithelial barrier function, suppressing pathogenic bacteria, and modulating host immunity (Dong et al., 2025; Tao et al., 2025). *Roseburia*, on the other hand, is a key butyrate-producing genus that supports mucosal integrity, inhibits intestinal inflammation, and promotes energy metabolism in colonocytes (Han et al., 2024a). These genera are closely associated with improved gut health and enhanced production of short-chain fatty acids, reflecting a gut environment more favorable for the proliferation of probiotics. This may be closely related to the enhanced antibacterial activity of mechanically activated zinc oxide against Gram-negative bacteria ([Bibr bib35][Bibr bib35]). Moreover, enhancement of intestinal barrier function plays a pivotal role in reducing bacterial translocation and systemic inflammation (Okumura and Takeda, 2024; Zhang et al., 2024). By preserving mucosal integrity, it restricts the translocation of gut bacteria and endotoxins into the circulation, thereby mitigating immune overactivation and maintaining systemic homeostasis. Notably, serum LPS and DAO are indicators of intestinal integrity and damage, which are closely associated with the inflammatory response (Kang et al., 2023; Wu et al., 2024). The results found that supplementing the diet with a low dose of mechanically activated zinc oxide significantly reduced serum LPS and DAO levels and increased anti-inflammatory cytokine (IL-10) contents on d 14 and reduced pro-inflammatory cytokines (IL-1β and IL-6) contents on d 28. Unexpectedly, serum IL-6 levels increased on d 14 following ZnO supplementation. The exact reason for this phenomenon is unclear, although it may be related to the specific pig breed or physiological stage used in this study. Further research is needed to verify this observation. These outcomes suggest that mechanically activated zinc oxide exerts beneficial effects on maintaining gut microbial balance and mucosal integrity (Zhi et al., 2023), thereby modulating the inflammatory response in weaned piglets as effectively as pharmacological ZnO but with reduced environmental risks.

The metabolomic profiling in this study reinforced the role of mechanically activated zinc oxide in modulating systemic metabolism, particularly through the tryptophan metabolic pathway. N-Acetylserotonin is a bioactive compound known for its antioxidant and anti-inflammatory effects (Kang et al., 2022). The elevation of N-acetylserotonin accompanied by the reduction of pro-oxidative quinoline derivatives has been associated with a beneficial metabolic transition, reflecting a systemic shift toward anti-inflammatory and tissue-repairing processes (Li et al., 2019; Savitz et al., 2022). It is consistent with the results of this experiment, N-acetylserotonin was positively correlated with CAT and SOD activities, and negatively correlated with BUN, LPS, and DAO levels. Such patterns suggest that N-acetylserotonin may participate in enhancing antioxidant defenses and preserving barrier integrity (Ben Shahar et al., 2016; Vašíček et al., 2020). In contrast, quinoline-4,8-diol and 4-(2-aminophenyl)-2,4-dioxobutanoic acid, both derived from alternative tryptophan catabolic pathways (Wang et al., 2024a), showed positive correlations with DAO and LPS, indicating their potential involvement in barrier disruption and oxidative stress. Furthermore, microbial–metabolite associations further support this interpretation. Specifically, N-acetylserotonin has strong positive associations with *Roseburia*, *Gemmiger*, *Ligilactobacillus*, and *Ruminococcus2*. These genera known for their anti-inflammatory or short-chain fatty acids (SCFAs)-producing capacities (Carbonne et al., 2023; Jiang et al., 2025; Ruan et al., 2022). Meanwhile, quinoline derivatives were negatively associated with these genera and positively linked to opportunistic pathogens like *Acinetobacter* and *Corynebacterium*. Taken together, these findings suggest that mechanically activated zinc oxide-mediated modulation of the tryptophan metabolism may be partly driven by microbial reshaping, reinforcing the microbial metabolite in promoting gut health. This suggests a hierarchical relationship where mechanically activated zinc oxide acts as the primary modulator of the gut microbiota, and the observed shifts in tryptophan metabolism are likely secondary consequences of this microbial optimization.

## 5 Conclusion

In summary, the present study revealed that dietary supplementation with mechanically activated zinc oxide at 400 mg Zn/kg improved growth performance and reduced diarrhea in weaned piglets, comparable to or better than 1600 mg Zn/kg ZnO. The protective mechanisms of mechanically activated zinc oxide against weaning stress in piglets involve the regulation of gut microbiota and tryptophan metabolism. Overall, these findings suggest that dietary supplementation with mechanically activated zinc oxide at 400 mg Zn/kg may be a feasible nutritional strategy as an alternative to high dose ZnO for improving the health of weaned piglets.

## CRediT authorship contribution statement

**Zhijie Yuan:** Writing – original draft, Methodology, Investigation, Formal analysis, Data curation. **Jingqi Huang:** Writing – original draft, Methodology, Investigation, Formal analysis. **Jinxin Guo:** Methodology, Investigation. **Bingbing Zhang:** Methodology, Investigation. **Weicheng Bei:** Resources. **Yong Wang:** Resources. **Yong Li:** Investigation. **Hairui Xin:** Investigation. **Weigang Xing:** Investigation. **Yakuan Huang:** Investigation. **Lvhui Sun:** Writing – review & editing, Supervision, Project administration, Funding acquisition, Conceptualization. **Zhangchao Deng:** Writing – review & editing, Visualization, Supervision, Project administration, Methodology, Formal analysis, Conceptualization.

## Declaration of competing interest

The authors declare the following financial interests/personal relationships which may be considered as potential competing interests: Zhijie Yuan, Hairui Xin, Weigang Xing, and Yakuan Huang are currently employed by Newhope Liuhe Co., Ltd., Beijing, China; Yong Li is currently employed by COFCO Feed Co., Ltd., Beijing, China; and Yong Wang is currently employed by Yishi Biotechnology Co., Ltd., Shanghai, China, which also funded this research project.
