# Supplementary material for: Mechanically activated zinc oxide enhances growth performance and protects against diarrhea with potentially regulating gut microbiota in weaned piglets
Source: Anim Nutr. 2026 Jun 27;26:652–64. doi: 10.1016/j.aninu.2026.03.002 (PMC13474652; doi:10.1016/j.aninu.2026.03.002)
Supplement: Supplementary file 1 [file mmc1.docx]

**Fig. S1** Relative abundance of four differential genus enriched in 400 Zn group of piglets at 28 d. BD, basal diet group; 400 Zn and 1600 Zn, BD supplemented with 400 mg Zn/kg mechanically activated zinc oxide and 1600 mg Zn/kg ZnO, respectively. Labeled means with different superscript letters are significantly different (*P* < 0.05).

**Fig. S2** Volcano plot analysis of the differential metabolites between BD and 1600 Zn groups (A, 14 d; C, 28 d). Volcano plot analysis of the differential metabolites between 400 Zn and 1600 Zn groups (B, 14 d; D, 28 d). BD, basal diet group; 400 Zn and 1600 Zn, BD supplemented with 400 mg Zn/kg of mechanically activated zinc oxide (MAXACTIVAT/Zn) and 1600 mg Zn/kg of ZnO, respectively. FC = fold change; VIP = variable importance in projection.

**Fig. S3** Kyoto Encyclopedia of Genes and Genomes (KEGG) pathway analysis of the serum differential metabolites between BD and 1600 Zn groups in piglets at 14 (A) and 28 d (C). KEGG pathway analysis of the serum differential metabolites between 400 Zn and 1600 Zn groups in piglets at 14 (B) and 28 d (D). BD, basal diet group; 400 Zn and 1600 Zn, BD supplemented with 400 mg Zn/kg of mechanically activated zinc oxide (MAXACTIVAT/Zn) and 1600 mg Zn/kg of ZnO, respectively. cGMP-PKG = cyclic guanosine monophosphate-protein kinase G; GABAergic = gamma-aminobutyric acid-ergic; FoxO = forkhead box O; PPAR = peroxisome proliferator-activated receptor; PI3K-Akt = phosphoinositide 3-kinase-protein kinase B; mTOR = mechanistic target of rapamycin; ABC transporters = ATP-binding cassette transporters.
